# Contextual Detection of Pedestrians and Vehicles in Orthophotography by Fusion of Deep Learning Algorithms

**DOI:** 10.3390/s22041381

**Published:** 2022-02-11

**Authors:** Masoomeh Shireen Ansarnia, Etienne Tisserand, Patrick Schweitzer, Mohamed Amine Zidane, Yves Berviller

**Affiliations:** Institut Jean Lamour (UMR7198), Université de Lorraine, 54052 Nancy, France; ansarnia1@univ-lorraine.fr (M.S.A.); etienne.tisserand@univ-lorraine.fr (E.T.); mohamed-amine.zidane@univ-lorraine.fr (M.A.Z.); yves.berviller@univ-lorraine.fr (Y.B.)

**Keywords:** deep learning, YOLO, semantic segmentation, FC-HarDNet, optical flow, FlowNet 2.0, orthophotography

## Abstract

In the context of smart cities, monitoring pedestrian and vehicle movements is essential to recognize abnormal events and prevent accidents. The proposed method in this work focuses on analyzing video streams captured from a vertically installed camera, and performing contextual road user detection. The final detection is based on the fusion of the outputs of three different convolutional neural networks. We are simultaneously interested in detecting road users, their motion, and their location respecting the static environment. We use YOLOv4 for object detection, FC-HarDNet for background semantic segmentation, and FlowNet 2.0 for motion detection. FC-HarDNet and YOLOv4 were retrained with our orthophotographs dataset. The last step involves a data fusion module. The presented results show that the method allows one to detect road users, identify the surfaces on which they move, quantify their apparent velocity, and estimate their actual velocity.

## 1. Introduction

The association of AI and video surveillance has been developing for years to provide citizens with new services [1,2,3]. Improving the safety of users of public roads, particularly those most vulnerable (pedestrians, cyclists), is one of the priority objectives [4,5]. This work examines the simultaneous use of three AI algorithms to analyze video streams delivered by a vertically installed camera in an urban area. The results of the algorithms are merged to obtain a contextual detection of the transient elements crossing the field of observation. These elements are mainly individuals or vehicles.

**Figure 1:**
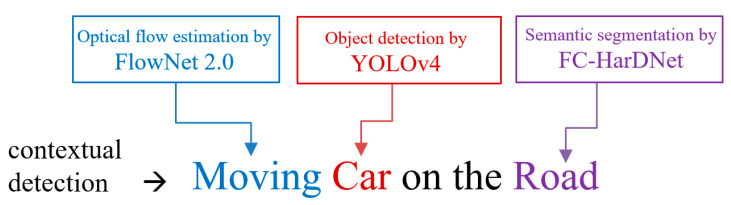
The concept of contextual detection in our project.

The concept of the contextual detection that we propose is presented in Figure 1. The goal and novelty of our work is the merging of the outputs of three algorithms to obtain a contextual detection related to a road user, as well as the position and motion of the road user. For instance, for a detected car driving on the road, the contextual detection provides the following three pieces of information: “moving”, “car”, and “road”.

The following applications that improve the cohabitation and circulation of road users are covered in this study:

- I2 V (infrastructure-to-vehicle) communication of the position and trajectory of users;
- Real-time and localized management of traffic lights according to the position of pedestrians and the density of traffic;
- Detection of infringement situations (e.g., vehicles parked on the sidewalk or lawn);
- Detection of dangerous situations (pedestrian outside a protected passage, immobilized vehicle, etc.).

To implement our project, the following three categories of algorithms are considered:

- Semantic segmentation, which identifies the main regions of interest on the ground (road, sidewalk, lawn, etc.);
- Object detection, to detect cars, pedestrians, cyclists, and road users in general;
- Estimation of the actual movement and velocity of the road users.

Developing architectures by stacking different networks into a large model is advantageous. For example, Mask RCNN [6] extends Faster R-CNN by adding a branch for predicting an object mask, in parallel with the existing branch for bounding box recognition. Hua et al. [7] also proposed instance segmentation by combining the function of semantic segmentation and object detection. 

Furthermore, other authors have developed networks that jointly perform the functions of apparent motion analysis and object detection. For example, reference [8] presents an association of YOLOv2 and FlowNet 2.0 for action detection, such as horse riding, skiing, etc. 

Another method for vehicle detection at far distances, and for velocity estimation, is proposed in [9], using Faster R-CNN and landmark-based scanlines on the road surface. FlowNet is also used for velocity direction estimation. This method measures the speed of vehicles with bidirectional movement, e.g., on highways, and does not apply to our problem since the objects can have any direction. The authors of [10] use YOLOv3 to reduce motion blur, video defocus, and partial occlusion, in optical flow estimated by FlowNet 2.0.

In [11], the authors use YOLOv2 for object detection and FlowNet for optical flow estimation. They also merge their results to estimate the speed of detected objects, without providing the direction of the velocity vector.

Despite more than 20 years of research in vision-based object speed estimation, and more than 135 papers analyzed, there remains an upward trend in the number of publications [12]. Among these, the fastest growing research focuses on traffic speed detection. Thus, the problem is still open, and there is no perfect solution at present. 

Our solution contains the following desirable features: the camera has a fixed location, but it can move slightly around its original position; the calibration is automatic; objects are detected, and their speed is estimated; the method applies to both traffic cameras and surveillance cameras; it can analyze the environment providing the type of road user, the path on which they move, their velocity and direction.

**Figure 2:**
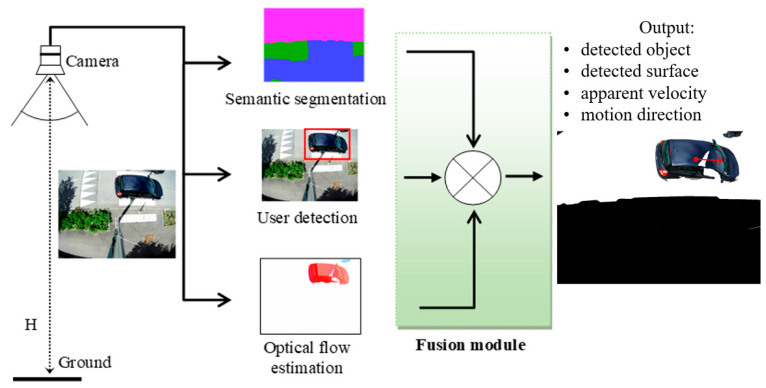
Illustration of the main objectives of the project.

Figure 2 depicts the guidelines of the project.

This work is not an end-to-end model intended to ensure the three functionalities mentioned in Figure 1. Instead, it uses three algorithms in parallel, where each is specialized in its field. A fourth module provides synthesizing and merging information from preceding networks. The latter is responsible for providing a situational and kinetic description of the road users.

The rest of this paper is structured as follows:

Section 2 presents the deep learning algorithms on which our study is based. Section 3 provides the materials and the conditions for obtaining videos of ortho-photographic urban scenes. Section 4 presents the results of the experimental campaigns carried out to test the performance of FC-HarDNet, YOLOv4, and FlowNet 2.0 individually for our particular configuration. The detailed fusion algorithm is provided in Section 5. Finally, in Section 6 and Section 7, this paper discusses our study’s contributions and debatable points, and suggests some improvements.

## 2. Related Works

### 2.1. Objects Detection

Many artificial intelligence models are dedicated to detecting objects, and recently this field has vastly improved due to the use of deep learning techniques [13]. Various versions of the YOLO [14] algorithm are among the most frequently mentioned in the literature, particularly the YOLOv4 [15] version, which is a state-of-the-art object detector that outperforms existing models in both detection performance and speed [16].

The architecture of YOLOv4 consists of CSPDarknet53 as the backbone, SPP and PAN models as the neck, and YOLOv3 as the head. It achieves up to 65.7 AP50 (average precision) on the COCO dataset with 80 classes, and higher if trained on fewer classes. As seen in Table 1, it possesses a well-balanced tradeoff between accuracy and inference time. 

Regarding the YOLO algorithm, our application implements the v4 version on RTX 8000 and the v4-Tiny version on a Jetson Nano.

### 2.2. Semantic Analysis of the Scene

Semantic segmentation is a computer vision task that can classify each pixel in an image [18,19]. It is commonly used in the fields of autonomous driving and robotics [20], satellite image analysis [21], and medical imaging [22].

To determine the position of objects in the scene, we require an interpreter of the environment. For this purpose, we have chosen the semantic segmentation approach, which is the task of associating a class to each photo pixel. The model used in this implementation is the fully convolutional (FC)-HarDNet for segmentation in PyTorch, and it is based on Harmonic DenseNet [23].

FC-HarDNet has a simple U-shaped encoder–decoder structure, only 1 × 1 or 3 × 3 convolutions, and no self-attention layer or pyramid pooling. It achieves 75.9% mIoU (*mean intersection over union)* on the Cityscapes dataset, the closest dataset to our input photos, e.g., images with the road surface, sidewalk, grass, lawn, etc.

Table 2 compares FC-HarDNet and the other segmentation models trained on the Cityscapes dataset. 

### 2.3. Estimation of Apparent Motion

Moving elements can be detected using background subtractor techniques [24,25]. These algorithms use a history of previous images to model the background and subtract it from the current image. They are economical in calculations; however, they do not quantify the movement’s speed or direction, and are subject to noise and error. Providing the apparent velocity requires more cumbersome methods, such as calculating the optical flow, which allows the estimation of each pixel’s motion vectors (U, V).

The analytic optical flow estimation requires hardware resources and is unfit for real-time applications. As an alternative, convolutional neural network solutions have appeared in recent years; for example, in 2015, Dosovtski et al. [26] proposed the FlowNetSimple and FlowNetCorr models to estimate the optical flow. The FlowNet 2.0 model, developed by Ilg et al. [27], derives from cascading several Simple and Corr models. A faster version of FlowNet was released in 2021 [28]. Another example is the PWC-Net model [29], which uses a pyramidal structure that allows the calculation of the flow to be densified using the “coarse-to-fine” approach recommended by the EpicFlow method [30]. Finally, the recurring RAFT [31] architecture was proposed by Teed and Deng in 2020.

Datasets, such as Kitty2005 and MPI Sintel, are used to benchmark optical flow estimation models. These datasets present many difficulties. For example, Kitty contains real dynamic scenes, including ample motions and variations in lighting, and MPI Sintel contains long scenes with blurred movements, reflection effects, defocus, and atmospheric disturbances.

## 3. Materials and Methods

### 3.1. Filming and Shooting Setup

To avoid obstacles in the field of view (FoV), the camera should be mounted at a sufficient height and tilted towards the ground. In practice, the angle of inclination is essential because it controls the perspective of the shooting and constitutes a parameter of primary importance for the 3D to 2D transformation. This parameter can vary depending on the surrounding structures (buildings, trees, etc.). We deliberately placed the camera vertically to eliminate the angular tilt parameter, regardless of the situation.

The chosen shooting configuration offers the following advantages:

- The FoV and the height ‘H’ of the camera are the only geometrical parameters of the installation;
- The area of interest is centered around the optical axis. For standard optics, the sky is absent from the images, limiting the risk of solar glare;
- The orthophotograph configuration allows easier horizontal plane segmentation and ground distance estimation;
- The location of an object in the horizontal plane is precise and simple to determine;
- The structures, such as advertising poles, lamp posts, and red lights, which are abundant in urban areas, are well-suited supports for fixing the camera;
- Detection of humans in this perspective could not be subject to mass surveillance, since facial features are practically impossible to recognize.

On the other hand, several drawbacks can be noted, for instance:

- With standard non-deforming lenses, the monitored scene area is small. For example, if a 90° FoV is used, the largest radius of the monitored area on the ground does not exceed H around its center. Using a wide-angle lens solves this problem at the cost of distortion of the images;
- Orthophotography offers minimal perspective, since objects are seen from above. In this case, distinguishing between pedestrians and bikes could be more challenging because their features are similar;
- The object detection algorithms have been mainly developed and trained on frontal shots. Their use in this context requires specific training.

### 3.2. Filming and Shooting Systems

**Figure 3:**
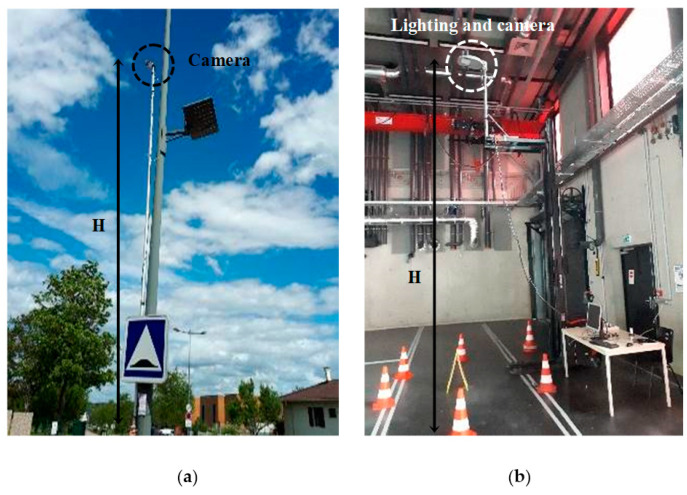
Diurnal (**a**) and nocturnal (**b**) test bench.

Daytime images were taken on public roads using a camera attached to the top of a 5.5 m mast (Figure 3a). Videos filmed by a drone at different altitudes were also used to create the dataset. For safety reasons, and to modify the lighting level, night images were taken indoors (Figure 3b). Lighting was provided by an adjustable LED light that delivered 8000 lumens at full power. The light and camera assembly were placed 5.4 m above the ground.

In both situations, we used an embedded camera with an Aptina AR0130 CMOS sensor and a 2.1 mm focal length lens that provided 98° horizontal FoV. The sensor provided a frame rate of 30 fps @ 1280 × 720 resolution or 15 fps @ 1280 × 960 resolution. In order to better quantify the detection performance under night conditions, some videos were filmed using a Canon EOS 2000D SLR in manual exposure mode.

### 3.3. Hardware and Software Development

Our algorithms were primarily developed in Python, tested on the Google Colab platform, and validated locally on an Nvidia RTX 8000 GPU. Real-time implementation capacities were evaluated on a NVIDIA Jetson Nano embedded GPU.

## 4. Individual Test of the Three Networks in Our Context

### 4.1. Semantic Segmentation of ROI (Region of Interest) on Ground

#### 4.1.1. FC-HarDNet Training and Validation 

The FC-HarDNet model, pre-trained on Cityscape, must be retrained on a personalized dataset containing orthophotographs specific to our application. To build this dataset, we used the mast-camera system shown in Figure 3a. The videos were filmed in six different places in a residential area in Nancy, France. In total, the length of the video footage is 6 min. From these videos, 120 images of resolution 1280 × 960 were extracted; 90 images were reserved for training and 30 for validation. Labeling was carried out with the LabelBox online tool. Six classes (car, road, sidewalk, vegetation, person, and bike) were selected for our application.

The training was achieved by transfer learning and using a Quadro RTX 8000 GPU. 

**Figure 4:**
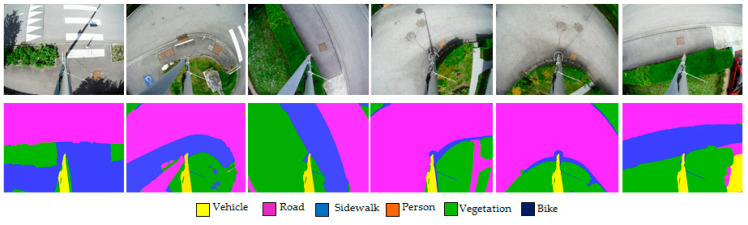
Segmentation of validation images.

Figure 4 shows some examples of the segmentation results of the validation images. One can observe the correct segmentations of the different ground surfaces, except for the metallic mast, which was always classified as a “Vehicle”. 

The mAP (*mean average precision*) and the mIoU values are summarized in Table 3. 

#### 4.1.2. Segmentation Results on Unseen Data

**Figure 5:**
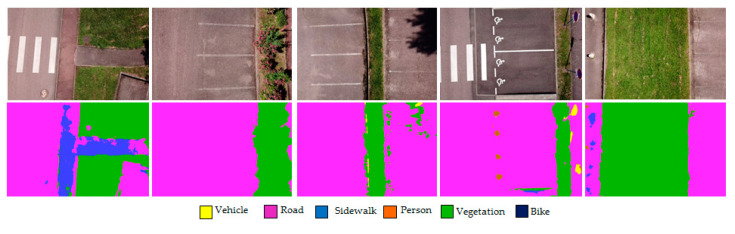
Segmentation of unseen orthophotographs, obtained with a drone at an altitude of 10 m.

We tested this model on orthophotographs of different outdoor locations (Figure 5).

These examples show that the system correctly detects asphalt and green spaces. On the other hand, a more substantial dataset will be necessary to train the network for roads and sidewalks. However, generally, the similarity between these two factors is deceiving for the model.

Subsequently, the Person, Vehicle, and Bike classes will be omitted from the output since these classes will be detected by the object detection model, as explained in the next section.

#### 4.1.3. Road User Segmentation

**Figure 6:**
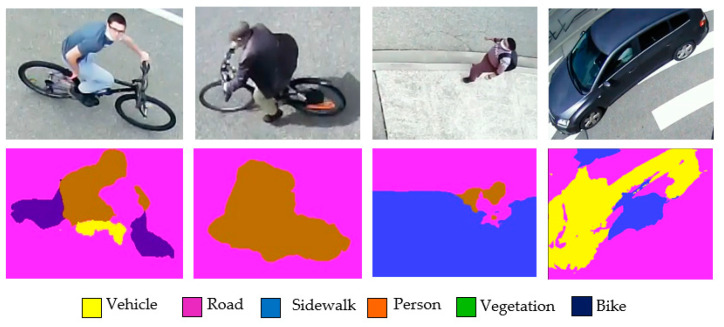
Typical cases of poor segmentation of road users with FC-HarDNet.

In this section, we study the ability of FC-HarDNet to correctly segment road users in an orthophoto image. The images in Figure 6 show some typical segmentation defects.

One can observe that the model occasionally struggles to detect road users of any type.

The primary defects found were:

- The distortion of the pedestrian’s silhouette while walking;
- Confusion between bikers and pedestrians;
- Incomplete segmentation of dark vehicles;
- Mixing labels when a user crosses the boundary between two ground areas.

This study shows that it is relatively challenging to employ FC-HarDNet alone to identify and locate all the static and non-static elements of the scenes. 

Indeed, we did not have a large dataset for training FC-HarDNet. The reason for this is that semantic segmentation requires demanding pixel-wise labeling, whereas creating an object detection dataset with distinct rectangular borders is less time-consuming.

### 4.2. Road User Detection

#### 4.2.1. YOLOv4 Training

In order to adapt YOLOv4 to the top-down view, especially at night, we conducted experiments before and after training the network on a series of videos under controlled lighting conditions. The illumination was fixed at eight lux, which is the typical minimum illumination requirement for roads in urban areas in France. 

We also used a DSLR camera to control the amount of light received by the sensor and to verify whether this impacts the precision of detection. The model was retrained on a custom dataset with approximately 10,000 photo samples, shot during night and day, including 8000 samples created with data augmentation [32]. The parameters used for training the model were the following: batch size = 64, image resolution = 416 × 416, learning rate = 0.001.

In addition, transfer learning was achieved using the pre-trained weights from the COCO dataset for better performance and faster training.

**Figure 7:**
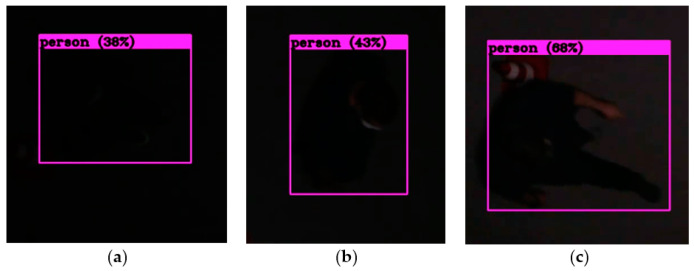
Pedestrian detection by YOLOv4 under the illumination of 8 lux. Exposure parameters: aperture = 3.5, shutter speed = 1/30, and ISO = 400 (**a**), 800 (**b**), and 1600 (**c**).

Figure 7 shows an example of detection by YOLOv4 of pedestrians under the illumination of eight lux.

#### 4.2.2. Detection Results 

A series of test videos were recorded by a DSLR camera to control the brightness of the footage by varying the ISO sensitivity. The algorithm was tested on these footages, and the results were compared using the Recall metric, since the priority was for detecting the maximum number of true positives. The aim was to verify whether a photos’ brightness significantly impacts the detection performances. 

The Recall metric is defined as:

(1) $$\mathrm{Recall}=\frac{\mathrm{True}\;\mathrm{Positives}}{\mathrm{True}\;\mathrm{Positives}+\mathrm{False}\;\mathrm{Negatives}}$$

Due to the low contrast of the images, we did not find any false positives in this experiment. Under these conditions, it is difficult to assess the precision metric.

Although the high percentage of Recall (Table 4) could be a sign of overfitting, in the context of this sensor, it is convenient because false detections are less detrimental, in our case, than missed detections.

**Figure 8:**
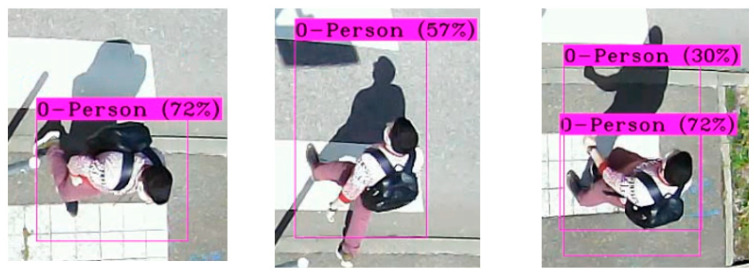
Inaccuracy of pedestrian detection in the presence of a shadow.

The detection results during the day show that the locations of pedestrians were less precise in the presence of a shadow, as shown in the example in Figure 8.

### 4.3. Optical Flow Estimation

We opt for the optical flow estimation by deep learning instead of tracking techniques for the following two reasons; firstly, the field of velocity vectors is determined over the entire image, which allows the highlighting of all moving elements.

Secondly, the estimation by FlowNet 2.0 is smoother, less noisy, and less resource-consuming than the analytical calculation of the dense flow. This approach seems to be well-suited to outdoor shots, the quality of which is strongly influenced by weather conditions.

**Figure 9:**
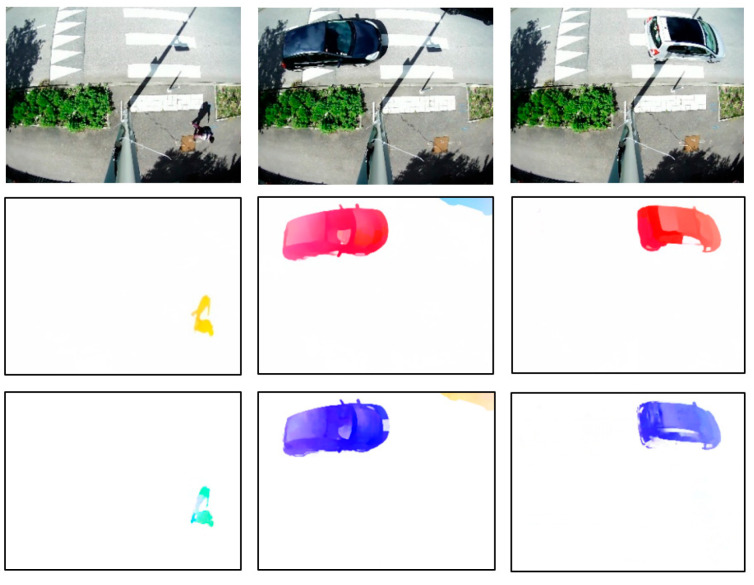
Examples of optical flow estimation with FlowNet 2.0 (**middle**) and PWC-Net **(bottom**), tested on our video sequences.

The FlowNet 2.0 and PWC-Net networks were tested on our video sequences as is. Some of the optical flow estimation results are shown in Figure 9. Note the different color palettes used by the two algorithms to represent the movement.

The movements of users (pedestrians and vehicles) appear with precision and with a minimal noise level. These satisfactory results show that it is unnecessary to conduct specific retraining with our dataset.

FlowNet 2.0 and PWC-Net showed similar performance, with a slightly smoother dense flow calculation for FlowNet 2.0. Thus, we selected this network for the rest of our experiments.

## 5. Fusion of Networks Results

### 5.1. Overall View of the Fusion Method

The main goal of the fusion process is to tag each bounding box of the detected objects with information regarding its motion and the region where it is located (Figure 1). Since the camera is stationary, the segmentation of the scene provided by FC-HarDNet can be static, i.e., it needs only to be carried out once. 

**Figure 10:**
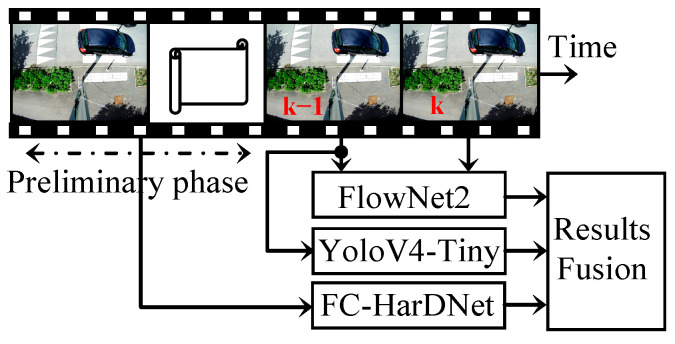
The overall structure of the fusion process.

On the other hand, YOLOv4 and FlowNet 2.0 process the video stream frame-by-frame. FlowNet 2.0 needs two consecutive frames to infer the motion field; thus, we decided to run the YOLOv4 inference with the second-to-last frame. The overall description of the fusion process is depicted in Figure 10.

### 5.2. Detailed Description of the Fusion Method 

As described in the previous paragraph, we worked with the two last frames as inputs for FlowNet 2.0 and the second-to-last frame as the input for YOLOv4. The FC-HarDNet output was a static pixel-wise segmented image with three classes (vegetation, sidewalk, road). The FlowNet 2.0 output was processed to split the U and V planes, compute the modulus of the velocity vector, and normalize it over the whole image between 0 and 255.

The fusion is performed by Algorithm 1.
**Algorithm 1. Fusion method.*****Require**:**Yolo: YOLOv4 inferred image of size NxM; FlowNet: FlowNet2 derived velocity magnitude image of size NxM; FC-HarDNet: HarDNet segmented image of size NxM****Ensure**:**List of Yolo detected objects along with their Mean Velocity and Segment of belonging**1: **for** BoundingBox ∈ Yolo **do****2:**MotionMask ← Binarize Flownet across BoundingBox using Otsu**3:****for** Vel ∈ MotionMask **do****4:*  *MeanVelocity ←*$\;\frac{\sum Vel}{MotionMask}$*5:****end for****6:**(Cx, Cy) ← center of gravity of MotionMask**7:****if** (Cx, Cy) ∈ Quarter1 **then****8:*  *Segment ← Hardnet(LowerLef t(BoundingBox))**9:****else if** (Cx, Cy) ∈ Quarter2 **then****10:*  *Segment ← Hardnet(LowerRight(BoundingBox))**11:****else if** (Cx, Cy) ∈ Quarter3 **then****12:*  *Segment ← Hardnet(U pperRight(BoundingBox))**13:****else****14:*  *Segment ← Hardnet(U pperLef t(BoundingBox))**15:****end if****16:**List ← append(BoundingBox, MeanVelocity, Segment)**17: **end for***

The fusion between the results of FlowNet 2.0 and YOLOv4 works as follows:

For each bonding box detected by YOLOv4, we binarize the motion field over the bounding box using the Otsu method [33] to obtain a motion mask.

The means of the motion field’s components, U_mean_ and V_mean_, are calculated across the unmasked region.

The motion mask’s center of gravity (C_X_, C_Y_) is also calculated.

Then, according to the center of gravity position, the algorithm decides which part of the bounding box corresponds to the base of the moving object, and identifies the region of the FC-HarDNet’s output to which the object’s base belongs.

**Figure 11:**
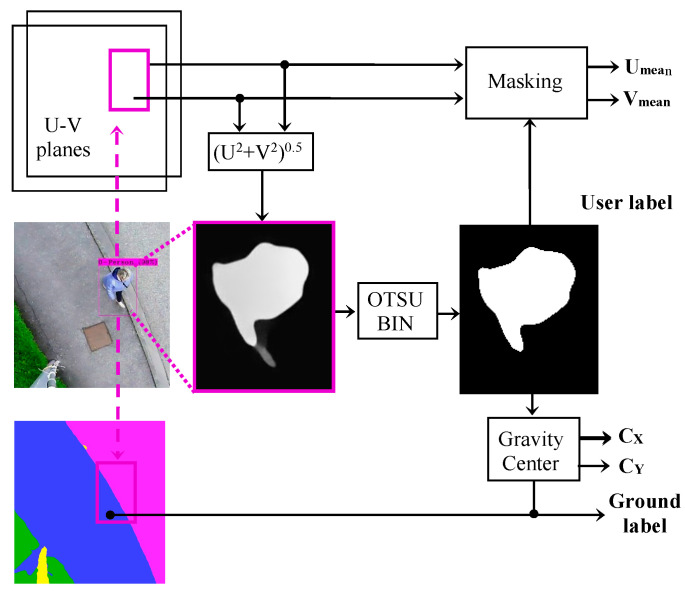
Block diagram of the fusion system.

This fusion method, represented in Figure 11, provides some additional benefits. For example, the Otsu binarization is greatly enhanced by the bounding boxes provided by YOLOv4, which ensures a bimodal (object/background) histogram of motion over a small area and not the entire frame. Furthermore, a small area reduces the risk of false optical flow detection e.g., wind-induced motion of grass or leaves, since YOLOv4 will not detect them.

**Figure 12:**
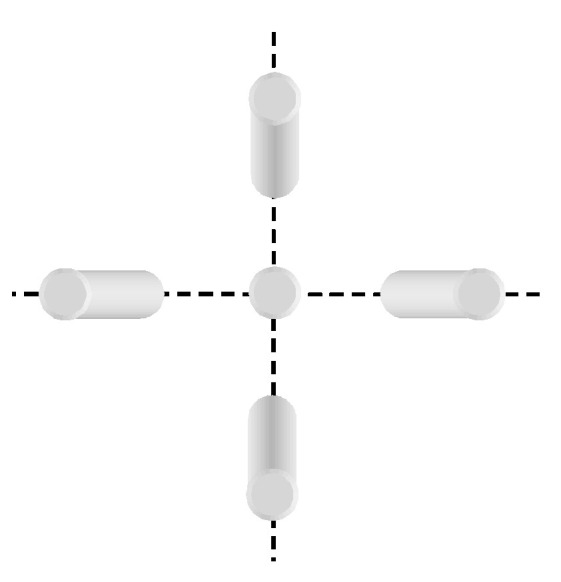
Location of the bottom of the detected objects.

Finally, the location of the base of objects is easily calculated due to the ortho-photographic setup. Using the quarter of the image that contains the center of gravity of the bounding box, we can determine which corner corresponds to the object’s base, as depicted in Figure 12 in the case of a cylinder object. For example, if the center of gravity coordinates are in the upper right quadrant, then the object’s base is close to the lowest, rightmost corner of the bounding box. 

### 5.3. Results

The fusion of the analyzes carried out by the three algorithms was tested on real urban scenes in which the user’s actual speed is not known. 

The shooting parameters used for the experiment are summarized in Table 5.

The origin of the pixel coordinates is located in the upper left corner of the images.

The optical magnification ‘g’ depends on the distance between the upper part of the object and the camera. For an object of height ‘h’, ‘g’ is calculated using Equation (2):

(2) g=f/(H−h)

where ‘h’ is estimated at 1.5 m for a pedestrian, a cyclist, or a car, which leads to g = 140 pixels/m.

**Figure 13:**
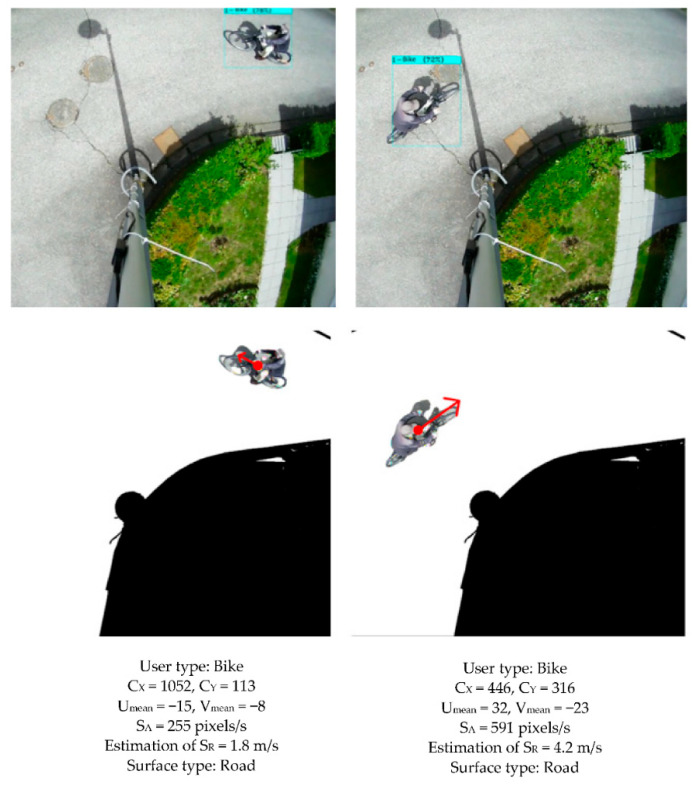
Detection, segmentation, and analysis of the movement of a cyclist. (**Top**): YOLOv4 detection. (**Center**): fusion of the three algorithms. (**Bottom**): quantified contextual information (type, positioning, and speed of the user, the nature of the surface).

**Figure 14:**
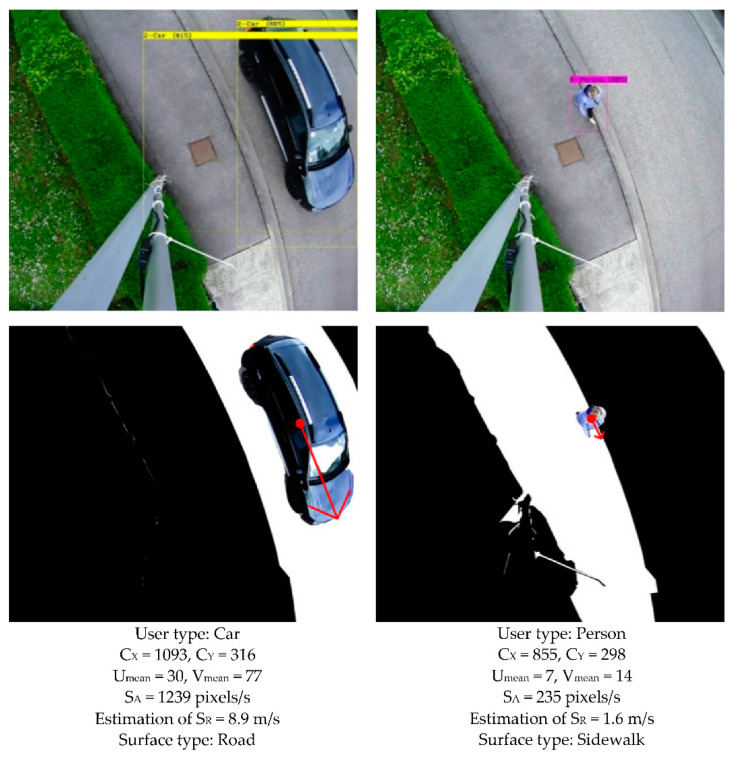
Detection, segmentation, and analysis of the movement of a car and a pedestrian. (**Top**): YOLOv4 detection. (**Center**): fusion of the three algorithms. (**Bottom**): quantified contextual information (type, positioning, and speed of the user, the nature of the surface).

Figure 13 and Figure 14 illustrate the results obtained when a cyclist, car, or pedestrian (at a brisk walk) crosses the field of view.

The surface upon which the user is moving is segmented and displayed in white. The other categories are eliminated (black). The beginning of the red arrow (point) indicates the position of the center of the gravity of the user. The arrow indicates the direction and modulus of the apparent average speed. The apparent speed ‘S_A_’ of the user is obtained by Equation (3), its actual speed ‘S_R_’ is estimated by Equation (4):

(3) $$S_{A}=\mathrm{FF}\times \sqrt{U_{\mathrm{mean}}^{2}+V_{\mathrm{mean}}^{2}}$$

(4) $$S_{R}=S_{A}/g$$

## 6. Discussion

### 6.1. Contributions of This Study

YOLOv4 is a reliable and accurate algorithm for performing road user detection; for this reason, it has a central place in our system. Furthermore, by selecting the analysis areas using bounding boxes, we reduce the risk of inaccuracy in FlowNet 2.0.

For each road user crossing the field of view, the algorithmic fusion extracts a vector characterized by six components: user label, the coordinates of the center of gravity in the image (C_X_, C_Y_), the apparent velocity (U_mean_, V_mean_), and ground label. Our system allows one to substitute each image with a matrix of anonymous metadata. This way, the privacy of the users and the confidentiality of the filmed locations is guaranteed. The delivered data allows for obtaining, simply and quickly, an instantaneous representation of the commuting and other human activities in the observed area.

The ortho-photographic images favor the perception of horizontal surfaces, which explains the satisfactory performance of FC-HarDNet in separating the different areas of interest on the ground. In addition, assuming a flat surface under the camera, the magnification factor remains constant during the movement of objects, which is not the case for perspective shots.

The method also applies to velocity estimation in fisheye shots, by introducing the projection model of the lens.

A list of the characteristics of “The contextual detection of urban users in orthophotography”, and other similar methods, is presented in Table 6. Although they are not designed to perform the same function, Table 6 summarizes the similarities and differences between the proposed model and other approaches.

The first difference between our proposed method and other methods, as shown in Table 6, is that we also provide the location of each object in the semantically segmented scene, providing contextual detection for urban area. The other difference between [11] and our method is that, instead of the k-means algorithm for object–background separation, we use the Otsu method. This method is fast compared to the k-means, which is slow for more extensive datasets, and provides an optimal threshold since it operates on a histogram.

Moreover, regarding the landmark-based scanline method proposed by Tran et al. [9], we propose placing the camera vertically to reduce the perspective so that the velocity estimation would be homogenous in the whole frame. 

### 6.2. Debatable Points

This study focuses on the qualitative results obtained during the field campaigns, as the multiplicity of possible situations does not allow us to establish generalizable quantitative results. Our objective is only to establish the proof of concept of the system. Training FC-HarDNet requires creating a more extensive database of labeled images. This step is time-consuming, and we plan to prepare it in the future.

We assume that the motion is uniform for all points of the moving element. This assumption is plausible for vehicles, but less so for pedestrians and cyclists, whose shapes alter during movement.

The first source of inaccuracy in the estimation of motion is related to general defects in the dense optical stream, i.e., aperture problems when objects enter or leave the observed area, or when these objects have low contrast. Secondly, the uncertainty in the mean height of moving objects, on which the optical magnification depends, distorts the estimates of the actual velocity. This uncertainty can be reduced by increasing the shooting height ‘H’.

Despite these inaccuracies, the proposed method allows a realistic estimation of the speed of objects in the observed scene.

## 7. Conclusions

The contextual detection of road users by fusion of the results of deep learning algorithms has been prepared and tested in this paper. Our multi-algorithmic approach makes it possible to provide important information about public road users, such as their type, position, velocity, and movement in a given environment.

The selected models are FC-HarDNet, YOLOv4, and FlowNet 2.0. The scenes are analyzed in the form of orthophotographs, which allows for locating users precisely on the horizontal plane and simplifies the relationship between the apparent speed, in terms of footage, and the actual speed.

In the future, priority improvements include:

- Applying the system to wide-angle lenses to expand the monitored area.
- Optimization for implementation on an embedded architecture.
- Calibration of the shooting system in order to quantify actual velocity.
- Extending of the dataset to ensure better training of YOLOv4 and FC-HarDNet.
- Verification of the detection performance under different weather conditions.

## Figures and Tables

**Table 1:** YOLOv4 performance [17].

Model	Backbone	Size (Pixel)	Inference Time (fps) on Pascal GPU	AP	AP_50_
YOLOv4	CSPDarknet-53	416	54	41	62.8
YOLOv4	CSPDarknet-53	512	43	43	64.9
YOLOv4	CSPDarknet-53	608	33	43.5	65.7
YOLOv4-Tiny	CSPDarknet-19	416	371	21.7	42

**Table 2:** Comparison of the performance of FC-HarDNet with other models.

Model	Inference Time (fps) on 1080ti @1024 × 2048	Cityscapes mIoU (%)
ICNet	48	69.5
SwiftNetRN-18	39.9	76
BiSeNet	27	77.7
**FC-HarDNet-70**	**53**	**76**

**Table 3:** Precision (mAP) and IoU of FC-HarDNet trained on Cityscape and on our personalized dataset.

Performances	Cityscape Dataset	Custom Dataset
General accuracy (%)	94	93.7
mAP (%)	84	86
mIoU (%)	76	79.5

**Table 4:** Recall for one class: Person.

ISO	Recall before Retraining	Recall after Retraining
400	17%	99%
800	21%	99%
1600	25%	99%

**Table 5:** Experimental conditions and optic parameters.

**Video resolution**	**Sensor sizes**	**Lens focal point ‘f’**
1280 × 960 pixels	4.8 × 3.6 mm	2.1 mm or 560 pixels
**Frame frequency FF**	**Camera height H**	**Optic magnification** **g**
15 fps	5.5 m	140 pixels/m

**Table 6:** Comparative specifications with other similar approaches.

Model	[8]	[9]	[11]	Proposed
**Objet detect. algorithm**	Yolov2	Faster RCNN-Resnet	Yolov2	Yolov4
**Speed detect. algorithm**	FlowNet 2.0	Landmark scanlines	FlowNet	FlowNet2
**Semantic Segmentation**	No	No	No	FC-HarDNet
**Dataset**	Kinetics	Nvidia AI City Challenge 2018	COCO and customized	Customized
**Video shooting mode**	Fixed perspective camera	Fixed perspective camera	Fixed perspective or mobile camera	Vertically installed, fixed camera
**Targeted application**	Human action detection	Vehicles on highways	Manless driving or Robot vision	Road users in urban area—various applications

## Data Availability

Not applicable.
